# Active vitamin D3 attenuates the severity of *Salmonella* colitis in mice by orchestrating innate immunity

**DOI:** 10.1002/iid3.408

**Published:** 2021-02-08

**Authors:** Fu‐Chen Huang, Shun‐Chen Huang

**Affiliations:** ^1^ Department of Pediatrics, Kaohsiung Chang Gung Memorial Hospital Chang Gung University College of Medicine Kaohsiung Taiwan; ^2^ Department of Pathology Kaohsiung Chang Gung Memorial Hospital Kaohsiung Taiwan

**Keywords:** colitis, innate immunity, *Salmonella*, vitamin D

## Abstract

**Introduction:**

*Salmonella* spp. pose major public health problems worldwide. A better understanding of the pathogenesis of these foodborne pathogens is a prerequisite for the design of improved intervention strategies that could reduce the use of antimicrobial agents and drug‐resistant Salmonellosis. Accumulating evidence indicates that vitamin D is involved in regulating innate immunity, and may, therefore, play a key role in human responses to infection. Studies have suggested 1,25‐dihydroxyvitamin D3 (1,25D3), the active form of vitamin D, effectively ameliorates colitis. These findings have broad implications for the use of vitamin D compounds in colitis. This study investigated the effect of active vitamin D3 on the severity of *Salmonella* colitis.

**Methods:**

A *Salmonella* colitis model was established with 6–8‐week‐old male C57BL/6 mice: Streptomycin‐pretreated C57BL/6 mice were infected orally with *Salmonella enterica* serova Typhimurium wild‐type strain SL1344 for 48 h. The mice were randomly assigned to control, model, and 1,25(OH)_2_D_3_‐treated groups. After the experiment, the mice were sacrificed, and intestinal, spleen, and liver tissue samples were removed to analyze bacterial colonization, western blot for protein levels, and real‐time‐polymer chain reaction for messenger RNA (mRNA) expression.

**Results:**

We observed that 1,25D3 reduced the severity of *Salmonella* colitis in C57BL/6 mice by reducing cecal mIL‐1beta, mIL‐6, mTNF‐alpha, and mIL‐8 mRNA expressions, bacterial colonization (CFU/mg tissue) in the liver and spleen, but increased the human β‐defensin‐2 mRNA and autophagy protein expression, compared to those of the SL1344 infection only.

**Conclusions:**

Our results document that active vitamin D3 reduced *Salmonella* colitis by decreasing inflammation, and bacterial translocation via induction of killing and autophagic clearance of pathogenic organisms.

## Graphical abstract

Our results suggest that active vitamin D3 reduced *Salmonella* colitis by decreasing inflammation and bacterial translocation via induction of killing and autophagic clearance of the pathogenic organism.

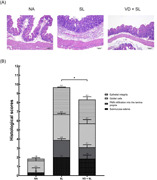

**Abbreviations**
1,25D3: 1,25‐dihydroxyvitamin D3
ATG16L1: autophagy‐related 16 like 1
hBD‐2: human β‐defensin‐2
IECs: intestinal epithelial cells
IL‐8: interleukin‐8
IL‐1β: interleukin‐1‐beta
NOD2: nucleotide‐binding oligomerization domain‐containing protein 2
ST: *Salmonella enterica* serovar Typhimurium
VDR: vitamin D receptor

## 1 INTRODUCTION

*Salmonella enterica* serovar Typhimurium (ST) is a major cause of bacterial enteric illness in humans. The incidence of foodborne human infections caused by *S. enteritidis* and multi‐drug‐resistant strains of ST has increased substantially in recent times with similar trends being reported in Europe and Taiwan.^1^ A better understanding of the pathogenesis of these foodborne pathogens is a prerequisite for the design of improved intervention strategies that could reduce the use of antimicrobial agents and drug‐resistant Salmonellosis.

*Salmonella* infection induces the intestinal mucosal epithelial cells to activate several signaling pathways leading to the production of inflammatory cytokines, chemokines, and antimicrobial peptides (AMPs).^2^, ^3^ The AMPs in *Salmonella*‐infected intestinal epithelial cells (IECs) may protect the host against infection, while modulation of pro‐inflammatory responses prevents the host from the detrimental effects of overwhelming inflammation.^4^

Recent evidence suggests that vitamin D enhances the innate immune response^5^, ^6^, ^7^ by induction of AMP including defensins and LL‐37 to exert antibacterial effects; while seeming to temper the inflammatory cascade induced by lipopolysaccharide (LPS). These peptides initiate bacterial killing by increasing bacterial cell membrane permeability once inside a phagosome. Clinically, lower 25(OH)D, defensin, and cathelicidin levels in sepsis patients were associated with severe illness as compared with those of the healthy controls.^8^

Several studies have suggested 1,25D3, was effective in ameliorating colitis through the regulation of innate immunity. Four studies were included in a systemic review^9^ regarding the therapeutic effect of vitamin D supplementation for colitis. Almost all the current data on how vitamin D can influence innate immune function stemmed from studies on human cells. A limited number of animal models have been utilized with varying results,^10^, ^11^ and further studies will need to improve upon this significantly.

Several studies have reported that autophagy plays an essential role in the host defense against various intracellular bacterial pathogens that use different strategies to establish infection, including *Listeria monocytogenes, Shigella flexneri*,^12^ and *Salmonella enterica* serovar Typhimurium.^13^ In addition, 1,25D3 induced the colocalization of mycobacterial phagosomes with autophagosomes in human alveolar macrophages in a nucleotide‐binding oligomerization domain‐containing protein 2 (NOD2)‐mediated cathelicidin‐dependent manner.^14^ These results strongly suggest that the stimulation of NOD2 expression in human epithelial cells by 1,25D3 increases autophagy, which then contributes to innate immune responses of vitamin D to bacterial infection.

Our in vitro studies^2^, ^3^ have demonstrated the effect of the differential regulation of active vitamin D on *Salmonella*‐induced cytokines and AMP, including interleukin‐8 (IL‐8), interleukin‐1‐beta (IL‐1β), and human β‐defensin‐2 (hBD‐2), and on autophagy expressions in the IECs with the involvement of different signaling proteins. Therefore, we investigated the immunomodulatory effects of active Vitamin D3 on *Salmonella* colitis in mice and the proteins involved.

## 2 MATERIALS AND METHODS

### 2.1 Bacterial strains

The naturally streptomycin‐resistant ST wild‐type strain SL1344 was grown for 12 h at 37°C in Luria–Bertani broth supplemented with 50 µg/ml streptomycin, diluted 1:100 in fresh broth, and subcultured overnight in static cultures with minimal aeration. The bacteria were collected using centrifugation at 14,000*g* for 5 min, washed with sterile phosphate‐buffered saline (PBS), and resuspended without antibiotics at a density of 4 × 10^9^ CFU/ml.

### 2.2 1,25(OH)_2_D_3_ treatment

Approximately, 50 ng of 1,25D3 was dissolved in 20 µl corn oil, and the mice were treated via oral gavage with 1,25D3 (0.2 μg/25 g mice) daily.

### 2.3 Animal experiments

A *Salmonella* colitis model was established with 6–8‐week‐old male C57BL/6 mice. Water and food were withdrawn 3 h before treatment with 20 mg streptomycin (100 μl sterile water for open control) per oral gavage. Then, the mice were supplied with water and food at will. Twenty‐four hours after the streptomycin treatment, food and water were withdrawn again for 3 h and the mice were infected with 10^8^ CFU (suspend in 100 μl PBS) of ST wild‐type strain SL1344. The food and water were supplied at will again. A total of 18 mice were randomly assigned to control, model, and 1,25D3‐treated groups (each group, *n* = 6). The mice in the 1,25D3‐treated group received 1,25D3 (Sigma‐Aldrich) daily (0.2 μg/25 g/day) in the diet via intragastric administration for 14 days, as described previously,^15^ along with ST infection, and the mice in the control and model groups were given PBS without and with ST infection, respectively (Supporting Information Figure). At the end of the experiment, the mice were sacrificed, tissue samples from the intestinal tracts, spleens, and livers were removed for analysis, and samples of the cecum were snap‐frozen in liquid nitrogen for the isolation of messenger RNA (mRNA) and proteins. Serum samples were collected for ELISA.

### 2.4 Histological colitis scoring

Postmortem, the entire colon was removed, from the cecum to the anus, and the colon length and weight were measured as markers of inflammation. Segments of the ileum, cecum, and colon were fixed and embedded in paraffin according to the standard procedures. Alternatively, tissue samples were embedded in optimal cutting temperature compound (Sakura Finetek USA Inc.), snap‐frozen in liquid nitrogen, and stored at −80°C. Part of the cecum was harvested and fixed in 10% formalin (pH 7.4), processed, and embedded in paraffin according to the standard protocol. Cryosections (5 μm) were mounted on glass slides, air‐dried for 2 h at room temperature, and stained with hematoxylin and eosin (H&E). Histological scoring was performed in a blinded fashion by a trained pathologist, with a combined score for submucosal edema (score, 0–3), polymorphonuclear granulocytes in the lamina propria (score, 0–4), number of goblet cells (score, 0–3), and epithelial integrity (score, 0–3).^16^ The combined pathological score for each tissue sample was determined as the sum of these scores; it ranges between 0 and 13 arbitrary units and covers the levels of inflammation in the intestine.

### 2.5 Analysis of ST loads in the spleens and livers

To analyze the colonization of bacteria, the spleens and livers were removed aseptically, weighed, and homogenized in 4°C cold PBS (0.5% Tergitol and 0.5% bovine serum albumin [BSA]) using a Potter homogenizer; a micro pestle was used to mince the tissue as much as possible. The numbers of colony‐forming unit (CFU) were determined by plating the appropriate dilutions on MacConkey agar plates (streptomycin at 50 µg/ml) for 16 h at 37°C under mild aeration. The minimal detectable values were 20 CFU/organ in the spleen and 100 CFU/organ in the liver.

### 2.6 Western blot analysis

Equal amounts of protein from the colon tissue were loaded into the wells of 8%–12% SDS–PAGE (sodium dodecyl sulphate–polyacrylamide gel electrophoresis) gels, electrophoresed, and transferred onto Immobilon P membranes (Millipore Corporation). After blocking the membranes with 3% BSA in Tris‐Buffered Saline and Tween 20 (TBST) at room temperature for 1 h, they were probed with primary antibodies to either anti‐autophagy‐related 16 like 1 (ATG16L1), anti‐LC3 (Cell Signaling Technology), NOD2 (Cayman Chemical), or anti‐vitamin D receptor (VDR; Cell Signaling Technology), and then developed with horseradish peroxidase‐conjugated secondary antibody (Cell Signaling Technology). Both antibodies were diluted in 5% skim milk (BD Biosciences) in 0.05% TBST (8 g NaCl, 0.2 g KCl, 3 g Tris, 0.5% Tween 20, double‐distilled H_2_O to 1 L, and pH 7.4). The filters were then stripped and re‐probed with antibodies to GAPDH (Santa Cruz Biotechnology) as appropriate. The chemiluminescent substrate was applied to the blot according to the manufacturer's recommendation. The protein bands were detected with the Bio‐Rad VersaDoc Image System (Bio‐Rad Laboratories Inc.). Protein expression was quantified via a densitometric analysis of the immunoblot images using the NIH ImageJ software (http://rsbweb.nih.gov/ij/). The results are expressed as relative intensity (mean ± *SEM*) compared to those of the normal controls.

### 2.7 Quantitative real‐time polymerase chain reaction (PCR) analysis of cecum RNA

Samples of the cecum were obtained, immediately snap‐frozen in liquid nitrogen, and stored at −80°C. Total RNA was extracted from the colon tissue in each group using TRI Reagent (#15596018; Ambion) and a Direct‐zol RNA MiniPrep Kit, according to the manufacturer's instructions. The reverse transcription step was performed using a TaKaRa PrimeScript™ RT reagent Kit (TaKaRaCat #RR037A; Takara Bio Inc.) in a 20 μl reaction volume with a final concentration of 1 μg total RNA, 5× PrimeScript buffer, 50 μM Oligo dT primer, 100 μM Random 6 mers, and PrimeScript RT Enzyme Mix1 per tube with RNase Free distilled H_2_O. Reverse transcription was performed in an ABI 2720 Thermal cycler (Applied Biosystems), programmed to run the reverse transcription for 15 min at 37°C, inactivate the reverse transcriptase for 5 s at 85°C and finally incubate the complementary DNA (cDNA) products at 4°C. Before the PCR step, the cDNA products were stored at −20°C.

Real‐time PCR (RT‐PCR) experiments were performed using the ABI 7500 Fast Real‐Time PCR System (Applied Biosystems). The reagent used was the Fast SYBR Green Master Mix (#4385612; Thermo Fisher Scientific) according to the manufacturer's instructions. The reaction mix contained 5 μl of sample mixed with 15 μl of PCR cocktail (200 nM for each primer). The primers (Genomics) for the genes of interest and the internal reference are as follows: Chemokine (C–X–C motif) ligand 2 (CXCL2, an analog of human IL‐8): forward, 5ʹ‐GCCCAGACAGAAGTCATAGCC‐3ʹ, reverse, 5ʹ‐GCTCCTCCTTTCCAGGTCAG‐3ʹ; Mouse beta defensin‐3 (mBD‐3, an analog of hBD‐2): forward, 5ʹ‐GCATTGGCAACACTCGTCAGA‐3ʹ, reverse, 5ʹ‐CGGGATCTTGGTCTTCTCTA‐3ʹ; Mouse interleukin‐1 beta (IL‐1β): forward, 5ʹ‐AGCTTCCTTGTGCAAGTGTC‐3ʹ, reverse, 5ʹ‐TTGGGGTCCGTCAACTTCAA‐3ʹ; Mouse tumor necrosis factor‐alpha (TNF‐α): 5ʹ‐CTCCAGGCGGTGCCTATGTC‐3ʹ, reverse, 5ʹ‐CCATTTGGGAACTTCTCATCCCTTT‐3ʹ, Mouse interleukin‐6 (IL‐6): forward, 5ʹ‐GTTCCTCTCTGCAAGAGACTTC‐3ʹ, reverse, 5ʹ‐AGTCTCCTCTCCGGACTTGT‐3ʹ, and Mouse beta‐actin (β‐actin): forward, 5ʹ‐TGTCGAGTCGCGTCCACC‐3ʹ, reverse, 5ʹ‐TCGTCATCCATGGCGAAC TGG‐3ʹ.

Each well was run in duplicate for the test primer and reference (β‐actin) and a duplicate sample set was run for each primer of interest. The reaction for each well was performed as follows: 95°C for 20 s, followed by 95°C for 3 s and 60°C for 30 s, and repeated for 40 cycles. The dissociation stage was performed as follows: 95°C for 15 s, 60°C for 60 s, 95°C for 15 s, and 60°C for 15 s. The ABI 7500 software (SDS V2.3) was used to obtain raw fluorescence data (Rn and DRn) for analysis. Many aspects of the MIQE guidelines were taken into consideration for the methods and analysis.^17^ The fold change in mRNA levels was determined using the comparative threshold cycle (CT) method (Applied Biosystems). Target gene transcription was normalized to the levels of β‐actin mRNA.

### 2.8 Statistical analysis

All the above experiments were performed in triplicate with similar results. The statistical analyses were performed using the paired Student's *t* test or Mann–Whitney *U* test for comparison of two parametric or nonparametric variables and Kruskal–Wallis one‐way analysis of variance for the comparison of nonparametric three or more variables (GraphPad Prism 8; GraphPad Software). *p* < .05 was considered statistically significant.

## 3 RESULTS

### 3.1 25D3 attenuates the severity of *Salmonella* colitis

**Figure 1:**
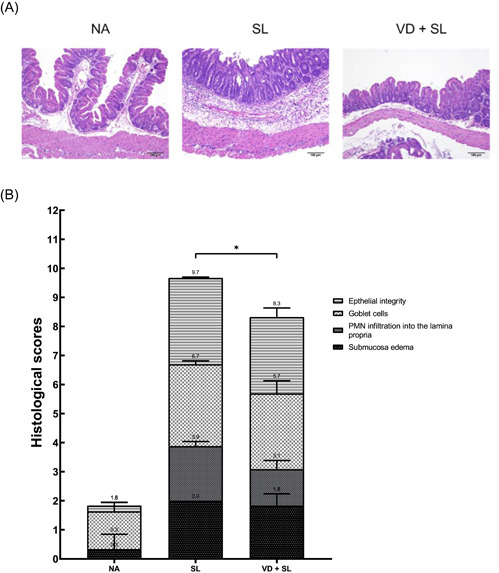
Active 1,25D3 attenuates *Salmonella*‐induced intestinal inflammation in mice. Female 6–8‐week‐old C57BL/6 mice (Charles River) were bred and housed under specific‐pathogen‐free conditions. The mice were orally administered with 20 mg of streptomycin, and incubated with PBS (NA) or 10^8^CFU of ST (SL1344 strain) for 48 h (ST). Mice receiving 1,25D3 (0.2 μg/25 g mouse) daily for 14 days along with infection acted as the 1,25D3‐treated group (VD + SL). Intestinal inflammation was evaluated 48 h later via (A) cecal histopathology (original magnification, ×100) and (B) histopathological score of cecal inflammation in mice. The scores were assessed using a combined score for submucosal edema (score, 0–3), polymorphonuclear granulocytes in the lamina propria (score, 0–4), number of goblet cells (score, 0–3), and epithelial integrity (score, 0–3), and a score of “13” showed the most significant level of disease pathology (*n* = 5). The results are depicted as mean ± *SEM*. *p < .05. 1,25D3, 1,25‐dihydroxyvitamin D3; CFU, colony‐forming unit; CA, control animal; PBS, phosphate‐buffered saline; PNM, polymorphonuclear; SL, SL1344 strain; ST, *Salmonella enterica* serovar Typhimurium; VD, vitamin D

To assess the effect of 1,25D3 on the severity of *Salmonella* colitis, we compared the cecal pathology of infected wild‐type (WT) mice with or without 1,25D3 treatment. Consistent with a previous study,^16^ the histopathological analysis of H&E‐stained cecum sections revealed obvious pathological changes in the ceca from the infected WT mice 48 h postinfection. In Figure 1A, we observed marked edema and infiltration of neutrophils in the submucosa, formation of crypt abscesses, desquamation in the surface epithelium layer, as well as the presence of necrotic epithelial cells in the ceca of these mice. In contrast, only mild inflammation in the ceca of the infected WT mice was observed in the 1,25D3‐treated ceca. These pathological features of inflamed ceca were absent in the uninfected mice. Using the pathological scoring scheme, we found that the severity of *Salmonella* colitis decreased significantly in the 1,25D3‐treated groups than in the WT mice (Figure 1B).

### 3.2 1,25D3 alters local inflammatory responses and AMP in the cecum of *Salmonella*‐infected mice

**Figure 2:**
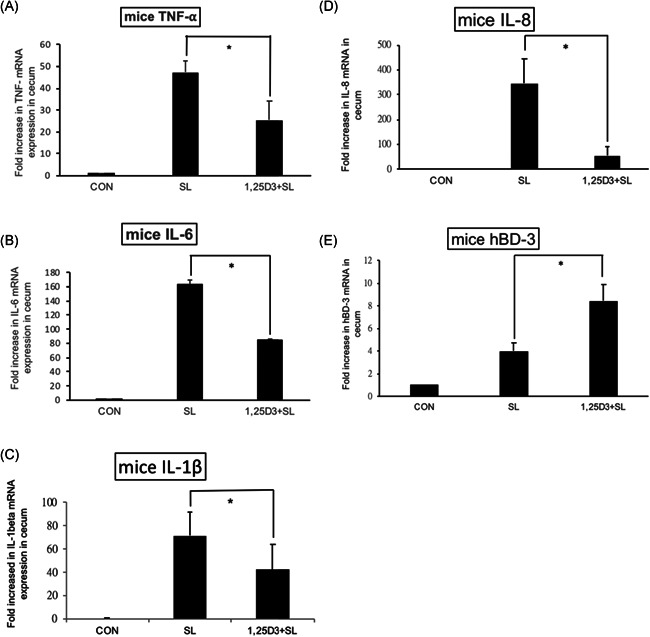
The immunomodulatory effects of 1,25D3 on cecal cytokines and antimicrobial peptide in *Salmonella* colitis mice. Female 6–8‐week‐old C57BL/6 mice (Charles River) were bred and housed under specific‐pathogen‐free conditions. The mice were orally administered with 20 mg of streptomycin, and incubated with PBS (CON) or 10^8^CFU of ST (SL1344 strain, SL) for 48 h, along with 1,25D3 treatment (0.2 μg/25 g mouse) daily for 14 days (1,25D3 + SL). Total RNA was extracted from the cecal tissues. mTNF‐α (A), mIL‐6 (B), mIL‐1β (C), mIL‐8 (D), and mBD‐3 (E) mRNA expressions were analyzed using quantitative RT‐PCR. The values were measured as fold increase compared to the levels in the control mice. The data shown are means ± *SEM* (*n* = 6 mice/group). An asterisk indicates the significant differences among groups, based on one‐way ANOVA. *p < .05. 1,25D3, 1,25‐dihydroxyvitamin D3; ANOVA, analysis of variance; hBD, human β‐defensin; IL, interleukin; mRNA, messenger RNA; TNF‐α, tumor necrosis factor‐alpha; PBS, phosphate‐buffered saline; RT‐PCR, real‐time polymerase chain reaction; ST, *Salmonella enterica* serovar Typhimurium

To confirm the in vitro effects of 1,25D3 on the inflammatory and AMP responses in *Salmonella*‐infected SW480 cells, we used the *Salmonella* colitis model. The expression of the cytokine and AMP genes was quantified using RT‐PCR on the cecal tissue of infected WT mice in the absence or presence of 1,25D3. Local, cecal expression of the cytokine and AMP genes (Figure 2) increased significantly in *Salmonella*‐infected mice, and the cytokines were markedly suppressed in the cecal tissue of *Salmonella*‐infected mice treated with 1,25D3, whereas the AMP increased significantly in the presence of 1,25D3.

We observed that 1,25D3 reduced cecal mTNF‐alpha (25.19 ± 8.72 vs. 47.28 ± 5.30, *p* < .05), mIL‐6 (84.422 ± 1.38 vs. 163.67 ± 5.63, *p* < .001), mIL‐1beta (42.01 ± 21.98 vs. 71.49 ± 20.38, *p* < .01), and mIL‐8 (53.70 ± 36.56 vs. 345.61 ± 99.33, *p* < .05) but increased mBD‐3 mRNA (8.43 ± 1.46 vs. 3.99 ± 0.72, *p* < .05) expressions.

### 3.3 1,25D3 reduced bacterial translocation in Salmonella‐infected mice

**Figure 3:**
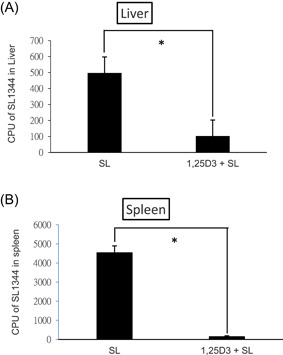
1,25D3 attenuates systemic bacterial translocation of *Salmonella colitis* mice. Female 6–8‐week‐old C57BL/6 mice were administered with streptomycin, and incubated with 10^8^ CFU of ST (SL1344 strain, SL) for 48 h, along with 1,25D3 daily for 14 days (1,25D3 + SL). The numbers of bacteria were counted from the liver (A) and spleen (B) homogenates of the control, *Salmonella*‐infected and 1,25D3‐treated mice at 48 h postinfection. The data shown are presented as means ± *SEM* of the bacterial load in the liver and spleen (*n* = 5–8). *p < .05. 1,25D3, 1,25‐dihydroxyvitamin D3; CFU, colony‐forming unit; ST, *Salmonella enterica* serovar Typhimurium

To determine the impact of 1,25D3 treatment on bacterial invasion, liver and spleen tissues were obtained from *Salmonella*‐infected mice treated with or without 1,25D3, homogenized, and plated on LB plates. The CFUs were determined. The results revealed that 1,25D3 treatment reduced bacterial loads in the liver or spleen of *Salmonella*‐infected mice (Figure 3).

We observed that 1,25D3 reduced bacterial colonization (CFU/g tissue) in the liver (1.02 ± 0.20 × 10^2^ vs. 4.97 ± 0.66 × 10^2^, *p* < .001) and the spleen (1.50 ± 0.42 × 10^2^ vs. 45.4 ± 3.56 × 10^2^, *p < *.0001), compared to that of the SL1344 infection only.

### 3.4 The effects of 1,25D3 on *Salmonella*‐induced autophagy and signaling protein expression in *Salmonella* colitis

**Figure 4:**
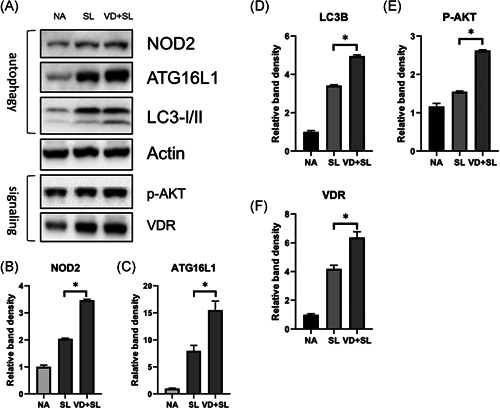
Effect of 1,25D3 treatment on the expression of cecal proteins in *Salmonella* colitis mice. Female 6–8‐week‐old C57BL/6 mice were administered with streptomycin, and incubated with PBS (NA) or 10^8^CFU of ST (SL1344 strain, SL) for 48 h, along with 1,25D3 (VD + SL) daily for 14 days. The cecum homogenates were used to assess the protein expression by immunoblotting with antibody to detect NOD2, Atg16L1, LC‐3B, VDR, and activated AKT (p‐AKT) proteins; actin was used for normalization of total protein. Representative immunoblots (A) and densitometric quantification of immunoreactive bands (B−F) are shown. The relative band intensities of NOD2 (B), Atg16L1 (C), LC‐3B (D), p‐AKT (E), and VDR (F) in protein extracts of cecal tissues in *Salmonella* colitis mice in the presence or absence of 1,25D3 were quantified as fold increases compared with those of the control mice. Data are expressed as mean ± *SEM* (*n* = 3 mice per group). *p < .05. 1,25D3, 1,25‐dihydroxyvitamin D3; Atg16L1, autophagy‐related 16 like 1; NOD2, nucleotide‐binding oligomerization domain‐containing protein 2; PBS, phosphate‐buffered saline; ST, *Salmonella enterica* serovar Typhimurium; VDR, vitamin D receptor

ST can induce colonic VDR expression and surface location in vivo, and stimulate VDR protein expression, transcriptional activity, and VDR‐mediated gene transcription in colonic epithelial cell lines.^18^ The activated autophagy of epithelial cells, depending on NOD2 and Atg16L1 expression, increased the killing of *Salmonella*.^19^ Additionally, our previous study^2^ demonstrated the effect of the differential regulation of 1,25D3 on *Salmonella*‐induced IL‐8 and hBD‐2 expression in IECs via PI3K/Akt signal and NOD2 protein expression, respectively, and active vitamin D increased the autophagic clearance of *Salmonella* infection while suppressing IL‐1‐beta mRNA expression via VDR and Atg16L1 protein response. It suggests PI3K/Akt signal, NOD2, Atg16L1, and VDR protein expression may act as a regulator for the immunoregulatory effects of 1,25D3 on local inflammatory responses and AMP in the cecum of *Salmonella* colitis. To examine the effects of 1,25D3 on *Salmonella*‐induced autophagy and the expression of signaling proteins, the western blots of PI3K/Akt, NOD2, Atg16L1, VDR, and LC‐3B proteins were analyzed in protein extracts of cecal tissues in *Salmonella* colitis mice in the presence or absence of 1,25D3. As shown in Figure 4, 1,25D3 increased the cecal NOD2, Atg16L1, and LC‐3B (autophagy), as well as VDR and p‐AKT (signaling) protein expression, compared to those of ST infection only.

## 4 DISCUSSION

Compatible with our in vitro study,^2^, ^3^ this in vivo investigation revealed that 1,25D3 reduced the severity of *Salmonella* colitis in C57BL/6 mice, reducing cecal mIL‐1beta, mIL‐6, mTNF‐alpha, and mIL‐8 mRNA expressions, and bacterial colonization in the liver and spleen, but increased the hBD‐2 mRNA. Recent research has increasingly unraveled the important roles of vitamin D in the regulation of innate immunity.^20^ In response to bacterial pathogens, AMPs are expressed at epithelial surfaces and are important effector molecules of innate immunity. The active form of vitamin D, 1,25D3, can significantly increase the production of AMPs in the urinary bladder epithelium during uropathogenic *Escherichia coli* infection^21^ to lessen susceptibility to urinary tract infection. Intestinal *Shigella* can turn off the endogenous expression of AMPs, resulting in serious shigellosis. Vitamin D may strengthen the mucosal defense by enhancing AMP expression in animal models of shigellosis as well as in clinical trials.^22^ Treatment with 1,25D3 has been reported to effectively protect against LPS‐induced disseminated intravascular coagulation in a rat model.^23^ Treatment of several cell lines or primary cell cultures with 1,25D3 induced the expression of two AMPs, hBD‐2, and cathelicidin AMP.^24^ AMPs disrupt the integrity of the bacterial cell membrane, resulting in the death of the pathogens including *Pseudomonas aeruginosa*.^24^ Human enterocytes contain β‐defensins that are secreted into the mucus layer to defend against bacterial transcytosis and act as chemoattractants for immune cells, in serving as a major innate defense against bacterial translocation, late‐onset sepsis, and necrotizing enterocolitis in preterm infants.^25^ This novel finding can be an alternative or complement to traditional antibiotics in the future.

A vitamin D response element is present in the proximal promoter of the human gene for hBD‐2,^24^ thus 1,25D3 is a direct inducer of its gene expression. Vitamin D (1,25D3) signaling induces NOD2 expression in the human IECs.^26^ Pretreatment with 1,25D3 to induce NOD2 expression, followed by NOD2 ligand muramyl dipeptide (MDP) synergistically induced hBD‐2 expression in the epithelial cells.^26^ We did demonstrate that 1,25D3 increased *Salmonella*‐induced cecal mBD‐3 mRNA expression in C57BL/6 mice. This regulation is essential for modulating the response to both NOD2 stimulation and infection with invasive intestinal pathogens, suggesting that it might play a critical role at the level of the intestinal mucosa.

Increasing evidence indicates the potential of autophagy in controlling infections by directing intracellular or ingested pathogens to lysosomes leading to their destruction.^12^, ^13^, ^27^ Invading/intracellular bacteria, with intact or even damaged membrane‐bound compartments, can be targeted by autophagy^28^ within the cytosol. To prevent bacterial escape, autophagy recognizes intracellular ST in damaged *Salmonella*‐containing vacuoles early after infection.^13^ Recent studies have linked the NOD2 function to autophagy.^29^, ^30^ The observation that *NOD2* is a 1,25D3 target gene also links vitamin D signaling to autophagy. In addition, 1,25D3 induced the colocalization of mycobacterial phagosomes with autophagosomes in human alveolar macrophages in a NOD2‐mediated cathelicidin‐dependent manner.^14^ Activated NOD2 recruits ATG16L1 to the cell membrane at the site of bacterial entry^30^ where ATG16L1 orchestrates protein complexes that control autophagy.^31^ These results strongly suggest that stimulation of NOD2 and ATG16L1 expressions in human epithelial cells by 1,25D3 would increase autophagy, which would then contribute to the innate immune responses of vitamin D to bacterial infection. We demonstrated that 1,25D3 increases the expression of autophagy proteins including NOD2, Atg16L1, and LC3‐II in the cecum of *Salmonella* colitis mice (Figure 4). Accordingly, the bacterial translocation to the liver and spleen decreased after 1,25D3 treatment (Figure 3). The regulation of NOD2 expression by 1,25D3 on downstream AMP production and autophagy contributes substantially to 1,25D3‐mediated innate antibacterial responses. This provides a strong molecular basis for the contribution of vitamin D deficiency to the pathogenesis of Crohn's disease (CD) because the interaction of *ATG16L1* and *NOD2* participate in an autophagy‐dependent antibacterial pathway implicated in CD pathogenesis.^19^, ^32^

Moreover, Atg16L1 has other unique protective functions, like negative regulation of pro‐inflammatory cytokine production,^33^ besides the release of an AMP. *ATG16L1* is a modulator of the balance between NOD2‐induced autophagy versus cytokine production. The *ATG16L1* 300Ala allele affecting ATG16L1 availability for complex formation with NOD2 could shift the balance of NOD2 downstream toward increased RIP2 signaling and elevated IL‐1β mRNA expression.^34^ TLR2‐ and TLR4‐induced signaling and IL‐1β production are not affected since TLRs have no physical interaction with ATG16L1. Lack of the ATG16L1 protein, upon NOD2 stimulation, displayed increased production of the pro‐inflammatory cytokines IL‐1β and IL‐6.^34^ Moreover, the inhibition of the autophagy process leads to increased pro‐inflammatory cytokine responses in human primary immune cells when stimulated with a ligand for NOD2. However, Atg16L1 may suppress the inflammatory cytokines induced by NOD2 in an autophagy‐independent manner.^35^ This study demonstrates that autophagy has a strong effect on the modulation of inflammation in vivo in humans, supporting the earlier murine and in vitro data. In humans, the effect of autophagy seems to be exerted mainly, if not exclusively, at the level of IL‐1β transcription. Besides this, activation of autophagy limits IL‐1β production by targeting ubiquitinated inflammasomes for destruction.^36^ Accordingly, we observed that 1,25D3 inhibited IL‐1β mRNA expression (Figure 2) though autophagy was increased. Perturbation of the NOD2–ATG16L1 axis, either in terms of autophagy induction or cytokine response, can contribute to the dysregulation of the mucosal immune response. It can explain our observation that vitamin D3 attenuated *Salmonella* colitis (Figure 1) while it augmented ATG16‐mediated autophagy (Figure 4).

VDRs negatively regulate bacteria‐induced NF‐κB activity in the gut and inhibit the downstream pro‐inflammatory responses, such as TNF‐α, IL‐1β, and IL‐6.^15^, ^18^ Mice lacking VDR activate the pro‐inflammatory NF‐κB pathway and are susceptible to *Salmonella*‐colitis and chemical‐induced colitis.^10^, ^18^ Furthermore, the treatment of IL‐10 KO mice with 1,25D3 resulted in the suppression of inflammatory bowel disease (IBD) symptoms.^11^ 1,25D3 upregulates the VDR through stabilization of the receptor in rat intestinal epithelial cells 6 (IEC‐6) ^37^ or the activation of gene expression in cells other than IECs.^38^ Clinically, oral supplementation with vitamin D3 significantly increased serum vitamin D levels and insignificantly reduced serum TNF‐α level in IBD patients.^39^ Vitamin D deficiency and impaired VDR signaling increased the levels of some pro‐inflammatory cytokines including tumor necrosis factor‐α (TNF‐α) and interferon‐γ.^40^ Moreover, studies in murine models are increasingly demonstrating that VDRs play a crucial role in attenuating colitis by regulating autophagy ^41^ and the production of AMPs.^18^ Accordingly, we observed the increased expression of VDR protein in mice with *Salmonella* colitis (Figure 4), which can explain the augmented autophagy and defensin expressions, as well as attenuated inflammatory responses. The methylation of the *VDR* gene affected its expression and the defense against pulmonary tuberculosis in human studies.^42^ In the future, studies in VDR knockout mice and epigenetic regulation of VDR will help to understand whether vitamin D increases antibacterial effects by a VDR‐dependent mechanism.

## 5 CONCLUSIONS

We demonstrated that 1,25D3 reduced *Salmonella* colitis by decreasing inflammation and reduced the bacterial load in the liver and spleen, indicating its immunomodulatory properties and protective role in the translocation of *Salmonella* and subsequent systemic infections, possibly through its regulatory effect on autophagy proteins and VDR. Thus, supplementation with vitamin D3 could provide a novel strategy to reduce antibiotic use among high consumers and indirectly prevent the emerging epidemic of bacterial resistance. The results from animal studies are of course limited in their application to the human species. Nonetheless, experimental models are an important foundation for establishing relationships between interventions and disease activity.

### 5.1 Limitations

The calcemic activity of 1,25D3 has limited its use in the treatment of conditions not related to mineral ion homeostasis. The therapeutic use of active vitamin D has been hampered by the toxic side effects of hypercalcemia. Fortunately, numerous analogs combine the more potent therapeutic activity with the weaker calcium mobilization.^43^ However, in the systemic review,^44^ no study showed that vitamin D or its analog supplementations are harmful to the participants or worsen their colitis. This indicates that they are both well‐tolerated. In contrast, there is an interest in developing vitamin D analogs that act as selective VDR ligands without inducing hypercalcemia. Finally, at the patient level, more clinical trials are needed to determine how vitamin D affects infection in vivo, and whether the levels of vitamin D required to do this are the same as those required for classical skeletal functions.

## CONFLICT OF INTERESTS

The authors declare that there are no conflict of interests.

## AUTHOR CONTRIBUTIONS

Fu‐Chen Huang designed the study, coordinated and supervised data collection, critically reviewed the manuscript, and approved the final manuscript as submitted. Shun‐Chen Huang reviewed the pathology, calculated histological scores, and coordinated and supervised data collection.

## ETHICS STATEMENT

Animal experiments were approved by the Institutional Animal Care and Use Committee (IACUC) in the Chang Gung Memorial Hospital authority and performed according to the legal requirements.

## Supporting information

Supporting information.

## Data Availability

The data that support the findings of this study are available from the corresponding author upon reasonable request.
