# Supplementary material for: Active vitamin D3 attenuates the severity of Salmonella colitis in mice by orchestrating innate immunity
Source: Immun Inflamm Dis. 2021 Feb 8;9(2):481–91. doi: 10.1002/iid3.408 (PMC8127544; doi:10.1002/iid3.408)
Supplement: Supplementary file 1 — Supporting information. [file IID3-9-481-s001.docx]

**Supplementary figure**

**Protocol for animal experiments** A *Salmonella* colitis model was established with 6–8 wk-old male C57BL/6 mice. Water and food were withdrawn 3 h before treatment with 20 mg streptomycin (100 μL sterile water for open control) per oral gavage. Twenty-four hours after the streptomycin treatment, food and water were withdrawn again for 3 h and the mice were infected with 10^8^ CFU (suspend in 100 μL PBS) of ST wild-type strain SL1344. The food and water were supplied at will again. The mice in the 1,25D3-treated group (VD+SL group) received 1,25D3 (Sigma-Aldrich, MO, USA) daily (0.2 μg/25 g/d) in the diet via intragastric administration for 14 days, along with ST infection, and the mice in the control (N/A) and model (ST) groups were given PBS without and with ST infection, respectively.
